# Glimpse into the genome sequence of a multidrug-resistant *Acinetobacter pittii* ST950 clinical isolate carrying the *bla*_OXA-72_ and *bla*_OXA-533_ genes in China

**DOI:** 10.1590/0074-02760170019

**Published:** 2017-10

**Authors:** Zhi Ruan, Yan Chen, Jianfeng Wang

**Affiliations:** 1 Zhejiang University, School of Medicine, Department of Clinical Laboratory, Sir Run Run Shaw Hospital, Hangzhou, Zhejiang, China; 2 The First Affiliated Hospital of Zhejiang Chinese Medical University, Department of General Practice, Hangzhou, Zhejiang, China; 3 The Affiliated Hospital of Hangzhou Normal University, Department of Respiratory Diseases, Hangzhou, Zhejiang, China

**Keywords:** Acinetobacter pittii, *bla*_OXA-72_, *bla*_OXA-533_

## Abstract

The development of carbapenem-resistant *Acinetobacter* species is of serious concern in the hospital settings and naturally occurring oxacillinase genes (*bla*_OXA_) have been identified in several *Acinetobacter* species. In this study, we report the genome sequence of *A. pittii* TCM178 belongs to ST950, a multidrug-resistant isolate that harbored the *bla*_OXA-72_ and *bla*_OXA-533_ genes simultaneous. The genome size was estimated to be 3,789,564 bp with 3,501 predicted coding regions, and G+C content is 37.60%. Our findings have raised awareness of the possible constitution of a reservoir for peculiar carbapenemase genes in *A. pittii* that may spread among other *Acinetobacter* species in China.

*Acinetobacter pittii*, formerly named *Acinetobacter genomic species* (*gen*. *sp*.) 3, is frequently associated with hospital-acquired infections and outbreaks that poses a particular concern due to its ability to acquire multidrug resistance to a wide range of antibiotics (Wang et al. 2014). Carbapenems have potent activity against *Acinetobacter* spp. and are often used as the last resort for the treatment of infections due to the high level of antimicrobial resistance. The resistance rates to carbapenems among *Acinetobacter* spp., mainly caused by intrinsically possessed an extensive arsenal of chromosomal genes encoding carbapenem-hydrolysing class D-lactamases (CHDLs), have increased dramatically in the last decade. To date, six phylogenetic subgroups of this enzyme have been identified in *Acinetobacter* spp., namely OXA-23, OXA-24/40, OXA-51, OXA-58, OXA-143 and OXA-235 (Evans & Amyes 2014). The gene *bla*_OXA-72_, one of the *bla*_OXA-40-like_ genes, was first identified in an *A. baumannii* strain isolated in Thailand, 2004. After that, this enzyme was reported in *Acinetobacter* spp. clinical isolates from different regions of the world. Genes encoding naturally occurring oxacillinases have been identified in several *Acinetobacter* species, such as *bla*_OXA-23-like_ (*A. radioresistens*), *bla*_OXA-51-like_ (*A. baumannii*), *bla*_OXA-134-like_ (*A. lwoffii/A. schindleri*), *bla*_OXA-211-like_ (*A. johnsonii*), *bla*_OXA-213-like_ (*A. pittii*/*A. calcoaceticus*), *bla*_OXA-214-like_ (*A. haemolyticus*) and *bla*_OXA-228-like_ (*A. bereziniae*), which could be utilised as a tool for rapid species identification (Montealegre et al. 2012, Kamolvit et al. 2015).

In the present study, we determined the whole genome sequence of a two OXA-type oxacillinases-producing *A. pittii* TCM178 isolate in China. The potential antibiotic resistance genes in TCM178 were predicted and a novel variant allele of the intrinsic *bla*_OXA-533_ gene has been found. These findings may improve our understanding of the antibiotic resistance mechanisms in *A. pittii*, and provide a clinical guidance for the therapy of *A. pittii* infections.

The strain *A. pittii* TCM178 was recovered from a blood sample of a male hospitalised patient with pneumonia at Hangzhou, Zhejiang province, China, in 2013. The strain was grown overnight at 37ºC in Mueller-Hinton broth (Oxoid, Hampshire, United Kingdom). It was identified according to *rpoB* gene sequencing analysis (La Scola et al. 2006). Antimicrobial susceptibility tests were conducted using the agar diffusion method and Etest (AB bioMérieux, Solna, Sweden), and interpreted according to Clinical and Laboratory Standards Institute (CLSI) 2017 guidelines (M100-S27).

Genomic DNA was extracted using a QIAamp DNA minikit (Qiagen, Valencia, CA) according to the protocol recommended by the manufacturer. Quality of the extracted DNA was examined using NanoDrop spectrophotometer (Thermo Scientific, Waltham, MA, USA) and a Qubit version 2.0 fluorometer (Life Technologies, Carlsbad, CA, USA). Subsequently, DNA library was prepared using Nextera™ DNA Sample Preparation kit (Nextera, USA), while the library quality was validated by Bioanalyzer 2100 high sensitivity DNA kit (Agilent Technologies, Palo Alto, CA) prior to sequencing. The DNA libraries with insert sizes of 300 bp were constructed from 5 μg DNA using an Illumina paired-end DNA sample prep kit (Illumina Inc., Cambridge, United Kingdom) and sequenced using the 2×150 bp paired-end sequencing strategy. The genome sequence of strain TCM178 was sequenced using an Illumina HiSeq 2500 platform (Illumina Inc., Madison, WI, USA). After sequencing, all sequence reads were preprocessed to remove low-quality or artifactual bases. The FastQC 0.11.5 was used for assessing the quality of the raw data, and the AlienTrimmer 0.4.0 was used to trim and discard the reads with a Phred quality score below 20. Finally, the fqduplicate 1.1 was used to discard every duplicate paired-end read. The trimmed reads were *de novo* assembled using CLC Genomics Workbench 9.0 (Qiagen, Valencia, CA). The optimal k-mer size was automatically determined using KmerGenie 1.7039. The contigs were linked and placed into scaffolds or supercontigs. The analysis was carried out using the SSPACE Premium scaffolder 2.3. The gapped regions within the scaffolds were partially closed in an automated manner using GapFiller 1.10.

The genome sequence was automatically annotated by the NCBI Prokaryotic Genomes Annotation Pipeline (PGAP) and Rapid Annotation using Subsystem Technology (RAST) server (Aziz et al. 2008, Seemann 2014). Classification of predicted proteins in clusters of orthologous groups (COG) functional categories was analysed with the WebMGA web server (Wu et al. 2011). PathogenFinder 1.1, ResFinder 2.1, CARD 2017, VirulenceFinder 1.5, PlasmidFinder 1.3, MLST 1.8 (MultiLocus Sequence Typing), and BacWGSTdb were used to estimate the number of pathogenicity determinants, acquired antibiotic resistance genes, virulence genes, plasmids and the sequence type (ST) using the assembled genome (Larsen et al. 2012, Zankari et al. 2012, Cosentino et al. 2013, Carattoli et al. 2014, Joensen et al. 2014, Ruan & Feng 2016, Jia et al. 2017). Minimum spanning tree (MSTree) analysis is a graph-based clustering method that can generate graphical results from MLST profile data. The PHYLOViZ 2.0 program can link the allele designations within the PubMLST database and draw an MSTree (Nascimento et al. 2017). The MSTree was calculated by Prim’s algorithm and was incorporated with the eBURST algorithm to obtain a comprehensive and precise graphical result. The graphical results included different types of lines were used to illustrate the number of shared alleles, and the length of branches reflect the distance between different types. Further bioinformatics analysis, such as identification of genomic islands, insertion elements (IS), prophage sequences, clustered regularly interspaced short palindromic repeat (CRISPR) sequences and secondary metabolite gene clusters, were predicted by application of IslandViewer 3, ISfinder 1.0, PHASTER 2016, CRISPRFinder 1.0 and antiSMASH 4.0.0 tools with default parameters, respectively (Siguier et al. 2006, Grissa et al. 2007, Dhillon et al. 2015, Weber et al. 2015, Arndt et al. 2016).

The draft genome sequence of *A. pittii* TCM178 consisted of 43 contigs which comprised 3,789,564 bases, and PGAP server predicted a total of 3,501 protein-coding sequences. The overall G+C content of this strain amounted to 37.60%. In total, 63 tRNA genes and 3 rRNA operons were identified, respectively (Fig. 1). The distribution of genes into COGs functional categories is summarised in Table. We also identified the aminoglycoside resistance genes *strA*, *strB* and *aph(3’)-VIa*, beta-lactam resistance genes *bla*_ADC-25_, *bla*_OXA-72_ and *bla*_OXA-213-like_, macrolide resistance genes *msr(E)* and *mph(E)*, and tetracycline resistance gene *tet(39)*. The allele of *bla*_OXA-213-like_ gene is a novel variant allele (271/273 identities) of intrinsic *bla*_OXA-533_ gene of *A. pittii* strain UKK_0145 (*bla*_OXA_ gene for OXA-213 family carbapenem-hydrolysing class D beta-lactamase OXA-533, complete CDS, NCBI Reference Sequence: NG_051483.1). The genome also contains at least 20 genomic islands and several IS elements: the majority belonging to the IS*3*, IS*5*, and IS*110* families. Similarly, there is one prophage sequence and three CRISPR sequences can be predicted in the genome. The presence of four putative secondary metabolite gene clusters, including the arylpolyne, bacteriocin, hserlactone and siderophore biosynthetic gene clusters can also be predicted. In addition, genome sequencing identified the gene *bla*_OXA-72_ was flanked by XerC/XerD-binding sites, a structure-specific recombination system implicated with its mobilisation. Upstream of the gene *bla*_OXA-533_, an N-acetyltransferase-encoding gene was identified; whereas the gene encoding a putative suppressor of F exclusion of phage T7 (*fxsA*) was found downstream. Genomic DNA digested with S1-nuclease and plasmid DNA from this strain were separated by PFGE, and subsequent Southern blot and hybridisation with *bla*_OXA_-specific probe showed that the *bla*_OXA-72_ gene was located on a plasmid of ~ 10 kb and the gene *bla*_OXA-533_ was located on the chromosome. The draft genome sequence of *A. pittii* TCM178 was also annotated using RAST server, with a total of 3,515 protein-coding sequences were determined, 2,141 of these were categorised into 451 subsystems, and 66 were RNAs. In the RAST-annotated genome, most of the genes were assigned into amino acids and derivatives (15.5%) followed by Carbohydrates (10.4%) and cofactors, vitamins, prosthetic groups and pigment (9.7%) subsystems. Genes encoding iron acquisition system, multidrug resistance efflux pumps and type IV secretion system were also identified in the genome. The MLST analysis showed that *A. pittii* TCM178 belongs to ST950, according to the MLST scheme of *A. baumannii* developed by Institute Pasteur. An MSTree algorithm was used to predict the clonal relationship and topological arrangement between *A. pittii* TCM178 and all 61 isolates in the PubMLST database. Only three clonal complexes were identified by the MSTree algorithm indicating limited genetic diversity among these isolates (Fig. 2).

**Fig. 1:**
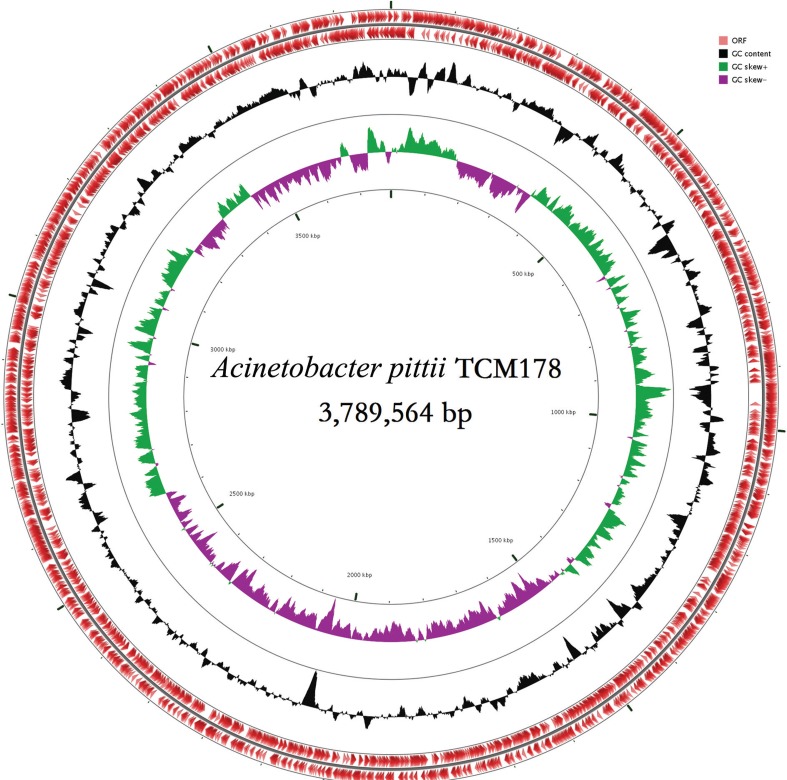
: graphical circular map of the genome of *Acinetobacter pittii* strain TCM178. The two inner circles indicate the G+C content plotted against the average G+C content of 37.60% (black circle) and GC skew information (green and purple circles). The outer circles display the open reading frames (ORFs) in opposite orientations.

**TABLE:** Number of genes associated with general clusters of orthologous groups (COG) functional categories

Code	Value	Total (%)^*^	Description
J	173	5.50	Translation, ribosomal structure and biogenesis
A	1	0.03	RNA processing and modification
K	278	8.84	Transcription
L	105	3.34	Replication, recombination and repair
B	1	0.03	Chromatin structure and dynamics
D	29	0.92	Cell cycle control, cell division, chromosome partitioning
V	34	1.08	Defense mechanisms
T	107	3.40	Signal transduction mechanisms
M	171	5.44	Cell wall/membrane/envelope biogenesis
N	41	1.30	Cell motility
U	87	2.77	Intracellular trafficking, secretion, and vesicular transport
O	114	3.62	Posttranslational modification, protein turnover, chaperones
C	200	6.36	Energy production and conversion
G	137	4.36	Carbohydrate transport and metabolism
E	295	9.38	Amino acid transport and metabolism
F	85	2.70	Nucleotide transport and metabolism
H	130	4.13	Coenzyme transport and metabolism
I	188	5.98	Lipid transport and metabolism
P	178	5.66	Inorganic ion transport and metabolism
Q	105	3.34	Secondary metabolites biosynthesis, transport and catabolism
R	397	12.62	General function prediction only
S	289	9.19	Function unknown
-	356	11.32	Not in COGs

*: the total is based on the total number of protein coding genes in the genome.

**Fig. 2:**
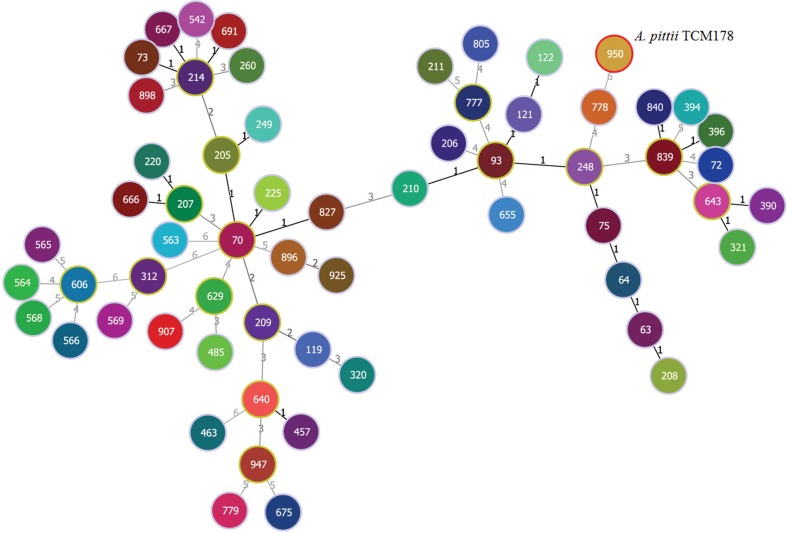
: minimum spanning tree analysis of *Acinetobacter pittii* isolates based on multilocus sequence typing (MLST) data. Each circle represents an independent sequence type (ST). The lines connecting the circles indicate the relationship between different STs. Different types of lines represent a difference in one allele (solid lines), two or more alleles (dashed lines). The numbers on the connecting lines illustrate the number of allelic differences.

In summary, these findings have raised awareness of the emergence of a multidrug-resistant ST950 *A. pittii* isolate producing OXA-72 and OXA-533 carbapenemases in China. The possible emergence of two OXA-type enzymes is worrying and must be monitored to avoid their major spread to more clinically relevant bacterial species. Further studies on the comparative genomic analysis of a large-scale sampling of *A. pittii* strains from a wide spatial and temporal range to get a more comprehensive picture of genomic epidemiological characteristics are warranted. To our knowledge, this is the first draft genome sequence of a multidrug-resistant OXA-72 and OXA-533 carbapenemase-producing *A. pittii* isolate in China.

The genome sequence of *A. pittii* TCM178 (Biosample ID: SAMN04452413) can be accessed at DDBJ/ENA/GenBank under the accession number LSAM00000000. The version described in this paper is the first version, LSAM01000000. The genome sequences data are available in FASTA, annotated GenBank flat file, graphical and ASN.1 formats.The genome project data are also available at GenBank under the genome Bioproject ID PRJNA310419.
